# Supplementary material for: Improved MPC for trajectory planning of self-driving cars
Source: PLoS One. 2025 Jun 3;20(6):e0320359. doi: 10.1371/journal.pone.0320359 (PMC12133195; doi:10.1371/journal.pone.0320359)
Supplement: Attachment — Submitted filename: Review Comments PONE-D-24-30241.docx [file pone.0320359.s001.docx]

**Review Comments PONE-D-24-30241**

**Summary of the Manuscript**

The manuscript presents an Improved Model Predictive Control (IMPC) approach for trajectory planning in self-driving cars. The study integrates vehicle state and driving context to effectively execute maneuvers, incorporating a simplified model of vehicle dynamics, a Sigmoid function to restrict movements, and a Finite State Machine (FSM) for real-time decision-making. The approach also utilizes a discrete Simplified Dual Neural Network (SDNN) to solve Quadratic Programming (QP) problems rapidly. The IMPC is validated through integrated Carsim/Simulink simulations, demonstrating effective collision avoidance in various driving scenarios. Some good qualities of the manuscript are as follows:

1. **Innovative Approach**: The integration of SDNN for rapid QP problem-solving addresses the slow-solving issues of traditional MPC, making the approach more efficient.
2. **Comprehensive Simulations**: The use of Carsim/Simulink simulations provides a robust validation of the proposed method across various driving scenarios.
3. **Clear Problem Definition**: The manuscript clearly outlines the challenges associated with lane changes in diverse driving environments and the need for improved control methods.
4. **Detailed Methodology**: The step-by-step explanation of the IMPC approach, including the use of a Sigmoid function and FSM decision-making, is thorough and easy to follow.
5. **Relevance**: The focus on collision avoidance and overtaking maneuvers in autonomous vehicles is highly relevant given the current advancements in vehicle technology and AI.

**Novelty and Contribution to the State of the Art**

- **Novelty**: The use of SDNN to solve QP problems rapidly and the integration of a Sigmoid function for trajectory constraints are novel contributions that enhance the efficiency and robustness of MPC in trajectory planning for self-driving cars.
- **Contribution**: The manuscript significantly contributes to the state of the art by addressing the computational efficiency issues of traditional MPC and providing a validated approach for real-time trajectory planning and collision avoidance in autonomous vehicles.

**Limitations of the Work**

1. **Limited Real-World Testing**: The study heavily relies on simulations, and there is a lack of real-world testing to validate the practical applicability of the IMPC approach. The validation through Carsim/Simulink simulations, while comprehensive, does not address potential issues that may arise in real-world implementations, such as sensor noise and unexpected obstacles. How does the proposed IMPC approach perform in real-world driving conditions compared to controlled simulations?
2. **Assumptions on Driving Scenarios**: The scenarios considered might not cover all possible real-world driving conditions, which could limit the generalizability of the results. The simplified model assumes ideal driving conditions which may not reflect the complexity of real-world scenarios, potentially affecting the accuracy of the proposed approach. Can the IMPC approach handle more complex driving scenarios involving multiple dynamic obstacles and varying road conditions?
3. **Computational Complexity**: Although the manuscript claims to address computational efficiency, the actual computational load of the proposed method in real-time applications is not thoroughly discussed. What are the computational requirements for implementing the IMPC in real-time on an autonomous vehicle platform?

**Points to Enhance the Quality of Work**

Some possible points which can be included to enhance the quality of work are as follows. If not within the scope of present work the authors should include these within the future scope of work:

1. **Incorporate Real-World Testing**: The authors may conduct experiments in real-world driving environments to validate the effectiveness and robustness of the IMPC approach.
2. **Expand Scenario Coverage**: The authors can include a wider range of driving scenarios in simulations to test the generalizability of the approach.
3. **Detail Computational Efficiency**: The authors may provide a more in-depth analysis of the computational requirements and efficiency of the IMPC approach in real-time applications.

**Grammatical Errors**

- **Sentence Structure**: Some sentences in the manuscript are lengthy and could benefit from restructuring for clarity. For example, the sentence "The selection of MPC [14] is justified for several reasons: MPC can integrate road boundary constraints, vehicle dynamic constraints, and passable area constraints into its framework" can be simplified for better readability.
- **Punctuation**: There are instances of missing commas which can affect the readability. For example, "Moreover a discrete Simplified Dual Neural Network (SDNN) is employed" should be "Moreover, a discrete Simplified Dual Neural Network (SDNN) is employed."
