# Supplementary material for: Improved MPC for trajectory planning of self-driving cars
Source: PLoS One. 2025 Jun 3;20(6):e0320359. doi: 10.1371/journal.pone.0320359 (PMC12133195; doi:10.1371/journal.pone.0320359)
Supplement: Attachment — Submitted filename: Response to Reviewers.docx [file pone.0320359.s003.docx]

Dear Editor and Reviewer,

Thank you very much for your comments and professional advice. These opinions help to improve the academic rigor of our article. Based on your suggestion and request, we have corrected the revised manuscript's modifications. We hope that our work can be improved again. Furthermore, we would like to show the details as follows:

**Journal Requirements:**

**Question 1: When submitting your revision, we need you to address these additional requirements.**

**Please ensure that your manuscript meets PLOS ONE's style requirements, including those for file naming.**

**The author’s answer:** Thank you for providing this suggestion. The manuscript has been modified according to the format provided on the PLOS ONE official website.

**Question 2: Please note that PLOS ONE has specific guidelines on code sharing for submissions in which author-generated code underpins the findings in the manuscript. In these cases, all author-generated code must be made available without restrictions upon publication of the work. Please review our guidelines at https://journals.plos.org/plosone/s/materials-and-software-sharing#loc-sharing-code and ensure that your code is shared in a way that follows best practice and facilitates reproducibility and reuse.**

**The author’s answer:** Thank you for your reminder. We have uploaded the program to Github. The link is: <https://github.com/s147896325/k.git>

**Question 3: Regarding the statement of funds**

**The author’s answer:** Thank you for your feedback. We have made modifications in the Acknowledgments Section and removed unnecessary content.

**Question 4: We note that your Data Availability Statement is currently as follows: All relevant data are within the manuscript and its Supporting Information files. Please confirm at this time whether or not your submission contains all raw data required to replicate the results of your study. Authors must share the “minimal data set” for their submission. PLOS defines the minimal data set to consist of the data required to replicate all study findings reported in the article, as well as related metadata and methods (https://journals.plos.org/plosone/s/data-availability#loc-minimal-data-set-definition).**

**The author’s answer:** Thank you for your feedback. We have submitted the data to figshare at link 10.6084/m9.figshare.28021790

**Question 5: PLOS requires an ORCID iD for the corresponding author in Editorial Manager on papers submitted after December 6th, 2016. Please ensure that you have an ORCID iD and that it is validated in Editorial Manager. To do this, go to ‘Update my Information’ (in the upper left-hand corner of the main menu), and click on the Fetch/Validate link next to the ORCID field. This will take you to the ORCID site and allow you to create a new iD or authenticate a pre-existing iD in Editorial Manager.**

**The author’s answer:** Thank you for your reminder. We have updated the ORCID ID of the corresponding author

**Question 6: Please review your reference list to ensure that it is complete and correct. If you have cited papers that have been retracted, please include the rationale for doing so in the manuscript text, or remove these references and replace them with relevant current references. Any changes to the reference list should be mentioned in the rebuttal letter that accompanies your revised manuscript. If you need to cite a retracted article, indicate the article’s retracted status in the References list and also include a citation and full reference for the retraction notice.**

**The author’s answer:** Thank you for your feedback. We have made revisions to the references in our manuscript based on the endnote style file on the reference format published on the PLOS ONE official website. If there are still some errors that we did not notice, we hope the editor can point them out for us. We would greatly appreciate it

**Reviewer 1**

**Question 1:Limited Real-World Testing: The study heavily relies on simulations, and there is a lack of real-world testing to validate the practical applicability of the IMPC approach. The validation through Carsim/Simulink simulations, while comprehensive, does not address potential issues that may arise in real-world implementations, such as sensor noise and unexpected obstacles. How does the proposed IMPC approach perform in real-world driving conditions compared to controlled simulations?**

**The author’s answer:** Thank you for your thorough review and insightful comments on our paper. As you have pointed out, the simulation environment is unable to fully replicate all variables encountered in real-world driving, especially the effects of sensor noise and dynamic obstacles in complex environments. In response, we have expanded our simulation validation to incorporate hardware-in-the-loop (HIL) experiments. The details of the HIL experiment are outlined as follows:

Through the HIL experiment, we further validated the efficacy of the proposed IMPC. The HIL setup comprised two industrial PCs (IPC 1 and IPC 2), a host computer, a target computer, a Controller Area Network (CAN), and SCANeR scene display software. IPC2 is equipped with SCANeR visual scene software to provide a simulated driving environment for the operator. The IPC is powered by a 2.8 GHz Intel Pentium Dual-Core E7400 processor and is equipped with 2GB of memory. Veristand-RT is employed to invoke individual submodules, establish connections between sub-signals, and deploy the model onto the target computer. The real-time version of CarSim-RT, provided by CarSim, operates synchronously with the target computer through frequency synchronization, closely approximating actual vehicle dynamics. CarSim-RT updates the vehicle's position, speed, and acceleration while displaying HIL simulation graphics. The target computer encodes the signals into CAN format for data transmission, and the CAN bus enables data exchange between the various platforms. This HIL experiment sets the control cycle to 10 ms, and the CAN bus baud rate is configured to 500 kbps. The hardware-in-the-loop simulation system is depicted in the figure below.

Hardware in the Loop Simulation System Diagram

A model diagram that can convert a '.m' file for QP solving into a '.C' file.

Signal Conversion Model Diagram

Below is the experimental result chart illustrating the driving scenario where the preceding vehicle is treated as an obstacle and a high-speed vehicle is traveling in the left lane.

The results of the HIL experiment are consistent with those of the joint simulation for the following reasons:

1. A delay was incorporated into the joint simulation to account for the time lag in data transmission during the HIL experiment.

2. White noise was deliberately added to the joint simulation to model the noise associated with CAN bus transmission.

Due to the limitations of our current laboratory equipment, we have not yet been able to perform the final validation on an actual vehicle. Nevertheless, preliminary results from the HIL experiment have demonstrated the feasibility of the IMPC method.
We greatly appreciate your thorough review and valuable suggestions. In response to your feedback, we have made the necessary revisions and additions to the paper.

**Question 2:Assumptions on Driving Scenarios: The scenarios considered might not cover all possible real-world driving conditions, which could limit the generalizability of the results. The simplified model assumes ideal driving conditions which may not reflect the complexity of real-world scenarios, potentially affecting the accuracy of the proposed approach. Can the IMPC approach handle more complex driving scenarios involving multiple dynamic obstacles and varying road conditions?**

**The author’s answer:** We greatly appreciate your thorough review and insightful feedback on our manuscript. In response to your comment on the "insufficient driving scenarios considered," we fully acknowledge your concern and offer the following detailed explanation.

This study primarily investigates the overtaking behavior of high-speed vehicles in same-direction dual-lane scenarios, to validate the feasibility of the IMPC method in these typical overtaking conditions. The study considers three primary scenarios: (1) the leading vehicle is an obstacle, with a high-speed vehicle in the left lane; (2) the leading vehicle is an obstacle, with the left lane unoccupied; and (3) the leading vehicle is an obstacle, with a low-speed vehicle in the left lane. These scenarios encompass the majority of typical high-speed, same-direction dual-lane driving situations encountered in real-world conditions.

Moreover, we have incorporated an experimental scenario involving overtaking and obstacle avoidance on a slippery road surface. Specifically, under normal driving conditions, the road friction coefficient is set at 0.85, and the road resistance coefficient at 1.0. In wet conditions, the friction coefficient is reduced to 0.65, and the road resistance coefficient is adjusted to 0.8.

Vehicle road surface parameter settings

Vehicle road resistance coefficient setting

The simulation results are as follows：

Based on the comprehensive experimental results, it can be seen that vehicles can avoid obstacles and overtake normally on wet and slippery roads, which proves the feasibility of IMPC.

**Question 3:Computational Complexity: Although the manuscript claims to address computational efficiency, the actual computational load of the proposed method in real-time applications is not thoroughly discussed. What are the computational requirements for implementing the IMPC in real time on an autonomous vehicle platform?**

**The author’s answer:** We sincerely appreciate your valuable feedback on our manuscript, particularly your insightful suggestions regarding computational efficiency. The issue of "computational complexity" that you raised is an aspect that we did not explain comprehensively in the original text, and we now have a deeper understanding of this concern. In response to your feedback, we have revised and expanded the manuscript accordingly.

Specifically, we compared the computational complexity between the traditional solution methods and those simplified via SDNN. In the revised manuscript, we have included a detailed discussion of the constraints in the traditional formulation versus those simplified using SDNN.
$\frac{\partial J_{QR,i}}{\partial_{\triangle u_{i}}}=2\left(\theta^{T}Q\theta+R\right)\triangle u+2E^{T}Q\theta\triangle u$

$$\kappa\frac{dz}{dt}=-KOz+(EO-I)g(z)-Kp$$

$$\bar{U}=Ov-p$$

Where$E$ and$K$ are known and$H$ is the symmetric matrix of quadratic programming, $z$ represents neurons, $g(z)$ denotes a nonlinear activation function, and $EO-I$is a symmetric weight coupling matrix.$O$and$s$ of the output equation$\bar{U}=Ov-p$ can be obtained offline. Through comparative analysis, we found that the SDNN method, in contrast to traditional approaches, eliminates matrix factorization and inversion operations, providing substantial computational advantages and lowering computational complexity. To illustrate this more clearly, we present in Figure (a) a comparison of computational efficiency under varying NP values, demonstrating that as the number of iterations increases, SDNN outperforms traditional methods in terms of computational efficiency, thereby emphasizing its computational advantage. Furthermore, in Figure (b), we provide a comparative analysis of SDNN and other existing algorithms under both single-input and multi-input conditions. These experimental results offer a comprehensive explanation of the superiority of the SDNN method in terms of solution speed, clearly demonstrating its potential for real-time applications.

|  |  |
| --- | --- |
| (a) Comparison between SDNN and Traditional Methods | (b) Comparison of different algorithms under different inputs |

The time limit for solving the algorithm is analyzed according to the following equation:

$$S_{f}=\frac{v_{x}^{2}}{2a_{e\max}}-\frac{v_{xi}^{2}}{2a_{i\max}}+v_{x}t+d_{0}$$

where$v_{x}$ represents the speed of the autonomous vehicle,$a_{e\max}$denotes its acceleration,$v_{xi}$signifies the speed of the obstacle vehicle,$a_{i\max}$indicates the obstacle vehicle's acceleration,$t$denotes the reaction time for peripheral information transmission, and$d_{0}$ denotes the minimum safe distance required. The longitudinal safety distance within the workshop and the maximum vehicle speed in this simulation indicate that the available reaction time for the vehicle is less than 1 second, encompassing the algorithm computation time, vehicle controller transmission, signal transmission, and other factors. The computation time for nine iterations of the SDNN remains within 150 ms, thereby ensuring that the solution efficiency of SDNN fully satisfies the requirements of this scenario.

**Question 4:Format issues and punctuation marks**

**The author’s answer:** Thank you for carefully reviewing our paper and providing valuable feedback on formatting, grammar, and punctuation. We sincerely apologize for the neglect of these issues and have made corresponding modifications and adjustments according to your suggestions. Specifically, we carefully examined the entire text. We corrected grammar errors, improper punctuation, and formatting inconsistencies to ensure the paper was more standardized and fluent in language expression and formatting.

**Review 2**

**Question 1:The point mass model employed in this study simplifies vehicle motion by disregarding dimensions and dynamics, such as load transfer during acceleration, braking, and turning. I understand the authors used this to reduce computational complexity, however, I’m wondering about its applicability in mixed traffic conditions, especially where vehicles of varying sizes and dynamics interact closely. Such interactions are common in diverse traffic environments, and ignoring these factors may lead to inaccuracies in predicting safe distances or potential collisions. Could you clarify how the point mass model accounts for or mitigates these potential inaccuracies, particularly in scenarios involving mixed and non-lane-based traffic, where diverse vehicle interactions are prevalent? Further, how was the model validated to ensure reliability in such complex traffic conditions?**

**The author’s answer:** The issues raised concerning the simplification of vehicle motion via the point mass model—such as the omission of dynamic factors like load transfer, acceleration, braking, and turning—along with the applicability of this simplification in mixed traffic conditions, are critical areas that warrant further investigation in our study. The point mass model reduces computational complexity, while the sigmoid barrier function constructs a safety barrier for each vehicle. This function automatically triggers braking or overtaking actions when vehicles approach, ensuring safe distances between them.

The safety distance, described by Equation$S_{f}=\frac{v_{x}^{2}}{2a_{e\max}}-\frac{v_{xi}^{2}}{2a_{i\max}}+v_{x}t+d_{0}$, considers the longitudinal length of 2.66 meters for the vehicles used in this simulation, along with a minimum longitudinal spacing of 1.3 meters in the workshop. The vehicle safety barriers are illustrated in the figure below.

Vehicle safety barrier

Therefore, although the point mass model neglects factors such as vehicle volume and mass, the introduction of the sigmoid barrier function allows vehicles to autonomously adjust their driving strategies in complex traffic environments, ensuring safety and reducing the risk of potential collisions.

For validation, before setting up the CarSim/Simulink co-simulation driving environment, we will test the reliability of the model by constructing a verification model (by selecting vehicles with different sizes and power characteristics for interaction in the CarSim vehicle selection interface).

Model validation

**Question 2：The omission of longitudinal and lateral load transfer effects may reduce the model’s accuracy in scenarios involving surrounding vehicles’ sharp turns, sudden braking, or acceleration, where these dynamics significantly influence vehicle performance. How does the model address scenarios require precise handling of dynamic effects, such as load transfer? Were any simplifications validated against real-world data to ensure their suitability for complex maneuvers?**

**The author’s answer:** Thank you for your valuable feedback on our research. In our model, we have adopted a simplified point mass model to describe the vehicle, to explore the effectiveness of vehicle control strategies. As you pointed out, neglecting the effects of longitudinal and lateral load transfer may affect the model's accuracy in certain extreme situations, such as during aggressive maneuvers. To address this issue, we have incorporated friction circle constraints when defining the boundary conditions, as shown in the figure.

Friction circle constraint

The resultant of the longitudinal and lateral forces in the point mass model should remain within the frictional limits that the road surface can provide. The coupling effect between the vehicle's lateral and longitudinal forces can be mapped to acceleration constraints through the tire friction circle constraint, ensuring that the combined lateral and longitudinal forces do not exceed the friction limits of the road surface, thereby preventing the vehicle from slipping or rolling over.

$$m\left[\left(a_{x}+a_{d}\right)^{2}+\left(a_{y}\right)^{2}\right]\leq\left(k\mu F_{z}\right)^{2}$$

$$\left\{\begin{matrix} F_{z1}=\frac{mgl_{r}}{2l}-\frac{ma_{x}}{2l}h-k_{f}\frac{ma_{y}}{d_{f}}h \\ F_{z2}=\frac{mgl_{r}}{2l}-\frac{ma_{x}}{2l}h+k_{f}\frac{ma_{y}}{d_{f}}h \\ F_{z3}=\frac{mgl_{f}}{2l}+\frac{ma_{x}}{2l}h-k_{r}\frac{ma_{y}}{d_{r}}h \\ F_{z4}=\frac{mgl_{f}}{2l}+\frac{ma_{x}}{2l}h+k_{r}\frac{ma_{y}}{d_{r}}h \end{matrix}\right.$$

The $F_{x}$ represents the longitudinal force, $F_{y}$ represents the lateral force, and $F_{z}$ represents the vertical load (dynamic load when the vehicle is in motion). In this context, $a_{d}$ refers to the acceleration due to the resistance encountered by the vehicle, $l_{r}=1.61m$ denotes the distance from the rear axle, $l_{f}=1.05m$denotes the distance from the front axle, $d_{f}=0.335m$ refers to the front track, $d_{r}=0.335m$ refers to the rear track, and $h=1.86m$ represents the vehicle height, the simulation vehicle has a mass of 1.4 tons, and the road surface friction coefficient is 0.85. The specific data are presented in the figure below:

vehicle parameters

We have imposed constraints on the vehicle's longitudinal and lateral accelerations using the friction circle, ensuring that they always remain within the tolerable limits of the road's adhesion. The allowable ranges of longitudinal and lateral accelerations are as follows:

$$\left\{\begin{matrix} a_{x\min}=-4\text{ }m/s^{2} \\ a_{x\max}=2\text{ }m/s^{2} \\ a_{y\min}=-2\text{ }m/s^{2} \\ a_{y\max}=-2\text{ }m/s^{2} \end{matrix}\right.$$

**Question 3:As real-world traffic scenarios often include challenges such as poor lighting, adverse weather conditions (while reading speed limits due to poor lighting), and unexpected obstacles (might not be of this study’s context), such as animals or pedestrians suddenly crossing the road. These factors can significantly impact the accuracy and reliability of motion predictions. In such a situation how does the model perform? Similarly, how does the model handle scenarios involving sudden, unpredictable events as mentioned earlier?**

**The author’s answer:** Thank you for your valuable feedback on our research. First, as outlined in Section 3 of the paper, the study assumes that the vehicle's speed and position, as well as those of surrounding obstacles, are known and that the road is straight. The effects of poor lighting and adverse weather conditions are part of sensor-based visual recognition and radar detection, which are beyond the scope of this paper. Regarding the impact of sudden obstacles on the study, while pedestrians are rarely encountered on highways, should a sudden obstacle appear, its position and speed can be determined, and the Sigmoid function can similarly provide a safety barrier, ensuring the safety of both the vehicle and the unexpected obstacle.

Once again, we appreciate your valuable suggestions. In future work, we also plan to investigate how such sudden events can be incorporated to enhance the model’s adaptability and reliability in more complex traffic scenarios.

**Question 4: The study utilized a decision-making framework for lane changes and braking based on predefined constraints and a finite state machine (FSM). Although this approach can be efficient, it may not be effective in addressing complex or rapidly changing traffic conditions, such as heavy congestion or unpredictable driver behavior. Is there any better adaptive decision-making mechanism (e.g., reinforcement learning or data-driven methods) to dynamically respond to varying traffic scenarios. Incorporating predictive modeling of surrounding vehicle behaviors could significantly improve safety and effectiveness.**

**The author’s answer:** Thank you for your important feedback on our research. First, regarding the suggestion to incorporate more flexible decision-making mechanisms, such as reinforcement learning (RL) or data-driven methods, we appreciate your input. We have indeed considered employing LSTM-MPC (Long Short-Term Memory Network-Model Predictive Control) to achieve dynamic decision-making, to address varied traffic scenarios. This approach presents several advantages: adaptability—RL can autonomously learn and adapt to complex and dynamic scenarios through continuous interaction with the environment, enabling it to manage large state spaces and uncertain conditions; no explicit rules—unlike finite state machines (FSM), RL does not require predefined state transition rules. Instead, it optimizes decision-making through a reward mechanism, making it suitable for handling incomplete or unknown information; flexibility—RL can address continuous, nonlinear, and high-dimensional decision problems, making it well-suited for complex environments such as autonomous driving.

However, the LSTM-MPC model presents challenges in practical applications, particularly concerning computational efficiency and obstacle avoidance, especially in highly dynamic and complex environments. The training and real-time inference of the LSTM model significantly increase computational load, which becomes a critical issue for real-time control systems. Consequently, we opted to implement a decision-making framework based on FSM. FSM offers the advantage of a simple structure and high computational efficiency, enabling stable operation in highway and relatively simple traffic scenarios. More importantly, FSM not only aids in decision-making but also shapes the subsequent Sigmoid function, which allows for the integration of decision outcomes with Model Predictive Control (MPC) for optimization. This approach effectively balances computational efficiency and obstacle avoidance. Specifically in our research scenario, FSM can flexibly respond to various traffic situations and optimize decision-making to ensure that the vehicle maintains efficient and safe operation in complex road environments.

In summary, the primary reason we chose FSM as the decision mechanism is due to its advantages in efficiency and interpretability. Moreover, it can be effectively integrated with MPC for optimization in practical applications. We sincerely appreciate your suggestion and will further elaborate on the strengths and scope of this decision framework in our paper, including discussions on its potential for extension and improvement in more complex traffic scenarios.

We tried our best to improve the manuscript and made some changes in the manuscript. Thank you for taking the time to review our paper. We look forward to receiving your reply。

Yours sincerely，

Kai Sun
